# Theaflavin-3,3′-Digallate from Black Tea Inhibits Neointima Formation Through Suppression of the PDGFRβ Pathway in Vascular Smooth Muscle Cells

**DOI:** 10.3389/fphar.2022.861319

**Published:** 2022-07-12

**Authors:** Yichen Wu, Min Chen, Zilong Chen, Jiangcheng Shu, Luoying Zhang, Jiong Hu, Hongjun Yu, Kai Huang, Minglu Liang

**Affiliations:** ^1^ Clinic Center of Human Gene Research, Union Hospital, Tongji Medical College, Huazhong University of Science and Technology, Wuhan, China; ^2^ Department of Cardiology, Union Hospital, Tongji Medical College, Huazhong University of Science and Technology, Wuhan, China; ^3^ Hubei Key Laboratory of Metabolic Abnormalities and Vascular Aging, Huazhong University of Science and Technology, Wuhan, China; ^4^ Hubei Clinical Research Center of Metabolic and Cardiovascular Disease, Wuhan, China; ^5^ Key Laboratory of Molecular Biophysics of the Ministry of Education, College of Life Science and Technology, Huazhong University of Science and Technology, Wuhan, China; ^6^ Department of Histology and Embryology School of Basic Medicine, Tongji Medical College Huazhong University of Science and Technology, Wuhan, China; ^7^ Department of Biochemistry and Molecular Biology, School of Basic Medicine and Tongji Medical College, Huazhong University of Science and Technology, Wuhan, China

**Keywords:** vascular smooth muscle cells, neointima formation, phenotypic switching, TF3, PDGFRβ signaling pathway

## Abstract

The abnormal neointima formation caused by the phenotypic switching of vascular smooth cells (VSMCs) into a synthetic state plays a key role in the pathogenesis of various vascular diseases, including atherosclerosis and postangioplasty restenosis. Theaflavin-3,3′-digallate (TF3) in black tea has been reported to exert antiinflammatory and anticancer effects, but its role in neointima formation remains unclear. Here, we delineated a remarkable effect of TF3 in suppressing neointima formation of VSMCs *in vivo* as well as the ability of primary rat aortic smooth cells (RASMCs) to proliferate and migrate *in vitro*. Further study confirmed that the effects of TF3 on PDGF-BB–induced RASMCs were due to reduced phosphorylation of PDGFRβ, which led to the repression of downstream pathways. We concluded that TF3 may act as a repressor in the progression of neointima formation and serve as a potential therapeutic candidate for excessive phenotypic switching of VSMCs.

## Introduction

Arteriosclerotic cardiovascular diseases (ASCVD), such as atherosclerosis, coronary heart disease, and postangioplasty restenosis (RS), are the main causes of mortality worldwide (Mortensen and Nordestgaard, 2018). Vascular remodeling resulting in an aberrant phenotype of medial smooth muscle cells is pervasively accepted as the primary pathological basis of ASCVD (Beach et al., 2013). In contrast to other mature cells, such as endothelial cells and fibroblasts, medial smooth muscle cells, which enable blood vessels to maintain structural and functional homeostasis under normal conditions, exhibit extensive plasticity to switch from a quiescent contractile phenotype to an active synthetic phenotype (Chistiakov et al., 2015). This aberrant transition in VSMC phenotype dramatically accelerates the pathogenesis of ASCVD (Chappell et al., 2016; Augstein et al., 2018), especially when induced by various pathogenic stimuli and stress signals, including vascular injury and mechanical force (Owens et al., 2004; Davis-Dusenbery et al., 2011; Heusch et al., 2014). For example, during the progression of postangioplasty restenosis (RS), injury-induced synthetic VSMCs express elevated proliferative factors and decreased contractile markers (Rensen et al., 2007; Frismantiene et al., 2018), migrate from the primary media to the intima, and secrete a large amount of extracellular matrix, leading to pathological thickening and vascular restenosis, which severely damage vessel functions.

The PDGFs are a number of cytokines belonging to the cystine knot protein superfamily. They exist as homo or heterodimers combined by four different monomers, namely, PDGF-A, PDGF-B, PDGF-C, and PDGF-D, and function as classical regulators of cell growth and cell division (Boor et al., 2014). PDGF-BB has been verified for its contribution to the formation of neointima after vascular injury (Raines, 2004; Tallquist and Kazlauskas, 2004; Shawky and Segar, 2017), and has become one of the most commonly used factors to induce fibroblast and VSMC proliferation and migration. Here, we used it to induce the synthetic phenotype of VSMCs *in vitro*.

Black tea, a fermented tea, provides multiple health benefits, such as refreshing effects and antiaging activity (Yang and Landau, 2000). The fermentation process of black tea results in the formation of theaflavins, comprising a mixture of theaflavin (TF1), theaflavin-3-gallate (TF2a), theaflavin-3′-gallate (TF2b), and theaflavin-3,3′-digallate (TF3), which is regarded as the most beneficial component of black tea (Liang et al., 1999). Previous studies have shown that theaflavins, especially TF3, can block nitric oxide synthase by downregulating NF-κB activation in macrophages (Lin et al., 1999). TF3 was recently reported to inhibit osteoclast formation and prevent ovariectomy-induced bone loss via suppression of the ERK pathway (Hu et al., 2017). Moreover, studies concerning the beneficial antioxidant, antiangiogenic, and antitumor effects of TF3 have also been published (Lin et al., 2000; Leung et al., 2001; Lee et al., 2004; Cai et al., 2007; Gao et al., 2016). However, whether TF3 can regulate the phenotypic switching of VSMCs remains unknown. Here, we elucidated the effect of TF3 on the regulation of injury and PDGF-BB–induced VSMC phenotypic switching and the underlying mechanisms.

## Materials and Methods

### Animals

C57BL/6 mice (8-weeks old) and male Sprague Dawley rats (170 g) were used in our experiments. The animals were housed on a 12:12-h light–dark cycle in a temperature-controlled and humidity-controlled room with free access to standard chow and tap water. All animal studies were approved by the Institutional Animal Care and Use Committee of Huazhong University of Science and Technology.

### Animal Experiments

The anesthetized C57BL/6 mice (*n* ≥ 6 for each group) were supine with their necks fully exposed and hair removed. First, the common carotid artery, the external carotid artery, and the internal carotid artery were carefully separated, and then the blood flow in a unilateral carotid artery was completely blocked using a ligature near the distal bifurcation. The same separation was performed for the carotid vessels on the other side as a sham operation. The mice were randomly injected intraperitoneally with normal saline (vehicle) or TF3 (10 mg/kg) the day before the operation and once every other day after ligation. Then, 14 and 28 days later, tissue sections were taken from the proximal end of the suture junction for histological tests.

### Histomorphometry

Tissue sections were fixed in 4% paraformaldehyde (PFA) and embedded in paraffin by Bios Biological Company. Serial 5-mm-thick transverse sections were stained in batches with hematoxylin and eosin and Masson to better differentiate intimal hyperplasia. Digital photographs were taken using an inverted microscope (OLYMPUS IX73). ImageJ software was used for morphometric analyses.

### Immunofluorescence

Slides of different sections were dewaxed in xylene, boiled for 10 min in citrate buffer for antigen retrieval, and exposed to blocking solution (5% goat serum in phosphate-buffered saline [PBS]) for 1 h at room temperature. PCNA (CST, #2586, 1:100 dilutions), MMP9 (Abcam, ab38898, 1:100 dilutions), α-SMA (Proteintech, 67735-1-lg, 1:100 dilutions), p-AKT (CST, #9231, 1:100 dilutions), and p-ERK1/2 (Abclonal, AP0974, 1:100 dilutions) were diluted in blocking solution and applied to the slides overnight at 4°C in a humidified chamber. The next day, slides were incubated with appropriate Alexa Fluor 488-, Alexa Fluor 555-conjugated secondary antibodies diluted 1:500 in blocking solution for 1 h at room temperature. DAPI (Servicebio, G1012) was then mounted on slides for 15 min. All immunofluorescence micrographs were taken using a confocal microscope (Nikon).

The cells were fixed in 4% PFA for 15 min at room temperature after reaching confluence, immunostained with α-SMA (Proteintech, 67735-1-lg, 1:100 dilutions) and SM22 (proteintech 10493-1-AP, 1:100 dilutions) antibodies overnight at 4°C, and then incubated with the indicated secondary antibodies for 1 h at 37°C. Nuclei were stained with DAPI for 20 min at 37°C. Photos were taken under a fluorescence microscope (Olympus).

### Cell Culture

For primary RASMC culture, the aorta of normal male rats was digested using collagenase II (Worthington) at a concentration of 2 mg/ml for 10 min, and then the adventitia was carefully stripped using microscopic tweezers. Endothelial cells were gently wiped off with a cotton swab. After overnight culture at 37°C in a 5% CO_2_ incubator in Dulbecco’s Modified Eagle Medium (DMEM) containing 10% FBS (Gibco), the remaining aorta was cut into pieces using microscissors and digested using collagenase II at 3 mg/ml for 30 min. Then, elastase (1 mg/ml, Worthington) was added for another 60 min. RASMCs were centrifuged, resuspended, and cultured in DMEM containing 10% FBS at 37°C in a 5% CO_2_ incubator. Primary mouse smooth muscle cells (mSMCs) were isolated in the same way. Then, 0.2 mg/ml collagenase II was used to remove the adventitia. After overnight culture, 1.0 mg/ml collagenase II and 0.1 mg/ml elastase were used for further digestion. All SMCs used for experiments were between the third and fifth passages.

### CCK-8 Assay

To estimate cell proliferation, RASMCs were seeded onto 96-well plates (5,000/well) and cultured in DMEM containing 10% FBS to near confluence. RASMCs were then cultured with FBS-free DMEM for 24 h and treated with PBS as a control or 20 ng/ml PDGF-BB (GenScript, Z03179) with or without adding different concentrations of TF3 (1, 10, and 20 μM) 1 h before exposure. The cells were incubated using CCK-8 for 2 h, and the absorbance at 450 nm was detected using a microplate reader (Multiskan FC, Thermo Fisher Scientific, Rockford, IL, United States).

### Western Blotting

RASMCs cultured in 6-well plates were starved in FBS-free DMEM for 24 h after nearly reaching confluence. The cells were pretreated with vehicle or different concentrations of TF3 1 h before stimulation. Total protein was extracted from the cells after 48 h, and the protein concentration was determined with a Pierce™ BCA protein assay kit (Thermo Scientific™, 23227), according to the manufacturer’s instructions. Then, western blot was conducted with primary antibodies against MYH11(Proteintech, 18569-1-AP, 1:1000 dilutions), α-SMA (Proteintech, 67735-1-lg, 1:1000 dilutions), SM22 (Proteintech, 10493-1-AP, 1:1000 dilutions), PCNA (CST, #2586, 1:1000 dilutions), cyclin E (CST, #20808, 1:1000 dilutions), CDK1 (Proteintech, 19532-1-AP, 1:1000 dilutions), CDK2 (Proteintech, 10122-1-AP, 1:1000 dilutions), CDK4 (Proteintech, 11026-1-AP, 1:1000 dilutions), MMP2 (Proteintech, 10373-2-AP, 1:1000 dilutions), MMP9 (Abcam, ab38898, 1:1000 dilutions), α-tubulin (Proteintech, 11224-1-AP, 1:1000 dilutions), GAPDH (Proteintech, 60004-1-lg, 1:1000 dilutions), p-ERK1/2 (CST, #5726, 1:1000 dilutions), p-JNK (CST, #4668, 1:1000 dilutions), p-p38 (CST, #9216, 1:1000 dilutions), ERK1/2 (ABclonal, A4782, 1:1000 dilutions), JNK (CST, #9252, 1:1000 dilutions), p38 (Proteintech, 14064-1-AP, 1:1000 dilutions), p-mTOR (CST, #5536, 1:1000 dilutions), mTOR (Bimake, A5866, 1:1000 dilutions), p-Akt (CST, #9271, 1:1000 dilutions), Akt (CST, #4691, 1:1000 dilutions), p-PDGF receptor beta (CST, #3161, 1:1000 dilutions), PDGF receptor beta (Bimake, A5541, 1:1000 dilutions), p-PLCγ1 (CST, #2821, 1:1000 dilutions), PLCγ1 (CST, #5690, 1:1000 dilutions), p-Src (CST, #6943, 1:1000 dilutions), and Src (CST, #2109, 1:1000 dilutions) at 4°C overnight. The blots were then incubated with secondary antibodies and visualized using ECL. ImageJ software was used for gray value analyses.

### EdU Assay

RASMCs were seeded onto 96-well plates (5,000/well) and cultured in DMEM containing 10% FBS until almost confluent. After starvation for 24 h, the cells were pretreated with vehicle or different concentrations of TF3 1 h before stimulation. Then, 48 h later, an EdU assay was performed following the standard protocol of the Cell-Light™ EdU Apollo®567 *in vitro* imaging kit (RiboBio, C10310-1). Photographs were taken using an inverted microscope (OLYMPUS IX73). ImageJ software was used for cell counting analyses.

### Wound Healing Migration Assay

RASMCs were seeded onto 6-well plates and grown to confluence. A straight line was scratched into the cells with a 200-μl pipette tip. The cells were pretreated with vehicle or different concentrations of TF3 1 h before stimulation. Then, they were allowed to migrate, and photographs from the same viewpoint were taken when the wound was made and 48 h later using an inverted microscope (OLYMPUS IX73). ImageJ software was used for wound area measurements.

### Transwell Assay

Flamed forceps were used to place the upper transwell chamber into a regular 24-well plate. Then, 100 µl of serum-free medium was added, and the chamber was placed back into the incubator for >1 h. Starved RASMCs were pretreated with vehicle or different concentrations of TF3 1 h before stimulation. The cells were incubated for 24 h, trypsinized, and resuspended in serum-free media so that 2 × 10^5^ cells were cultured in 100 μl of medium. Then, 500 µl of fully supplemented medium (containing 10% FBS) was added to the lower chamber. Flamed forceps were used to transfer the upper chambers above the lower chamber. Care was taken to avoid trapping air bubbles below the surface of the membrane. The cell suspension was immediately added to each of the upper chambers followed by incubation for another 24 h. Then, the medium was aspirated from the upper chamber, and the inserts were transferred into another well containing PBS for washing. The inside of the upper chamber was scraped with a cotton swab to remove the cells from the inside of the well. Cells on the underside of the insert were stained by placing the insert into crystal violet solution for 15 min, and photos were taken using an inverted microscope (OLYMPUS IX73). ImageJ software was used for cell counting analyses.

### Statistical Analysis

The data are expressed as the mean ± SEM values. The Shapiro–Wilk tests and two-tailed unpaired students’ tests were used to determine significance of differences between two groups. Statistical significance was indicated by *p* values < 0.05.

## Results

### TF3 Attenuates Carotid Artery Ligation–Induced Neointimal Hyperplasia

**FIGURE 1:**
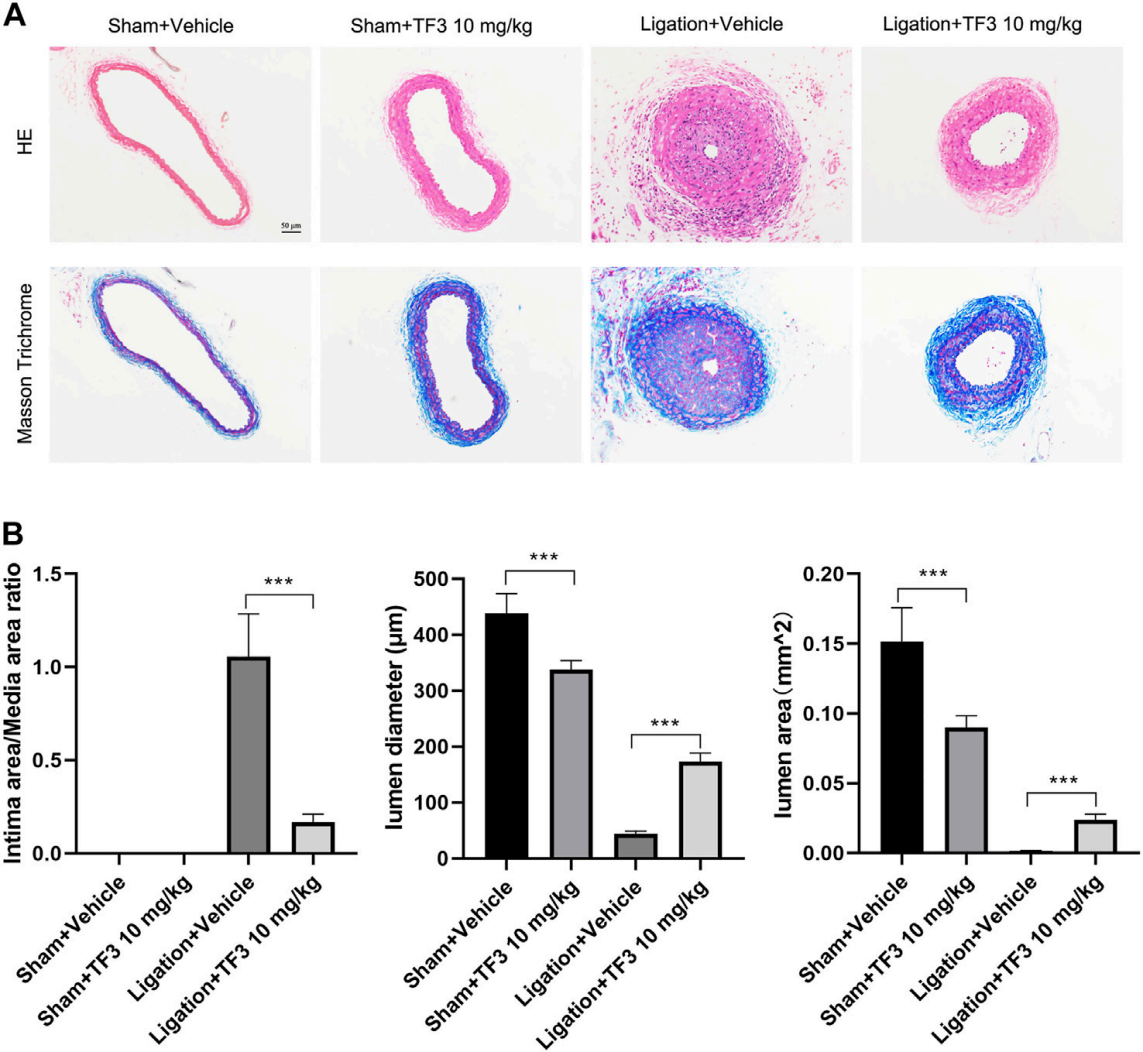
Effect of theaflavin-3,3′-digallate (TF3) on carotid artery ligation–induced neointimal hyperplasia. **(A)** TF3 (10 mg/kg) or vehicle was intraperitoneally injected after carotid artery ligation. Mice were sacrificed 14 days after the surgery. Hematoxylin and eosin‐stained and Masson trichrome‐stained sections of all groups are shown. Scale bar, 50 μm. **(B)** Quantification of the ratio of intima to media (I/M ratio), lumen diameter, and lumen area. The data are presented as the mean ± SEM (*n* = 7). ***p < 0.001.

**FIGURE 2:**
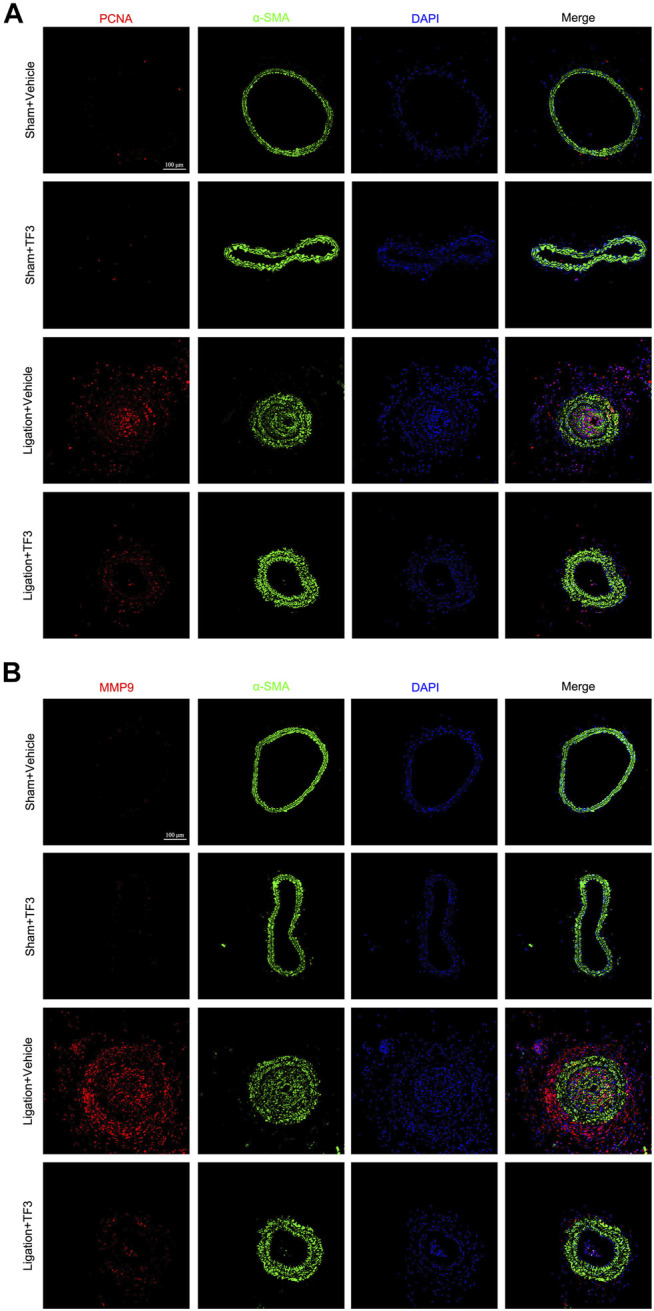
Effect of theaflavin-3,3′-digallate (TF3) on carotid artery ligation–induced neointimal hyperplasia. TF3 (10 mg/kg) or vehicle was intraperitoneally injected after carotid artery ligation. **(A)** Immunofluorescence staining of PCNA (red), α-SMA (green), and DAPI (blue) are shown. **(B)** Immunofluorescence staining of MMP9 (red), α-SMA (green), and DAPI (blue) are shown. Scale bar, 100 μm.

To assess the effect of TF3 on neointimal hyperplasia after vascular injury, we used the carotid artery ligation (CAL) model, which gave rise to the progression of vascular restenosis. TF3 (10 mg/kg) or vehicle was intraperitoneally injected one day before CAL and repeated every other day. Compared with the vehicle-treated sham surgery group, the vehicle-treated ligation surgery group showed well-developed neointimal hyperplasia after 14 days. The sections stained with HE and Masson trichrome solutions were used to highlight the media (Figure 1A). Compared with the vehicle-treated ligation surgery group, the TF3-treated ligation surgery group showed a prominently reduced ratio of intima to media (I/M ratio) with apparently larger lumen diameter and lumen area (Figure 1B). There was also a significant difference between the two sham surgery groups of vehicle- and TF3-treated mice in lumen sizes due to their different levels of compensatory dilatation. Immunofluorescence staining showed that the TF3-treated ligation surgery group had sharply declined PCNA and MMP9 expression compared to vehicle-treated surgery group (Figure 2). HE and Masson staining were also performed 28 days after surgery (Supplementary Figure S1) as well as immunofluorescence assay (Supplementary Figure S2). These results indicate that TF3 significantly attenuates neointimal hyperplasia induced by CAL.

### TF3 Attenuates PDGF-BB–Induced Phenotypic Switching in RASMCs

**FIGURE 3:**
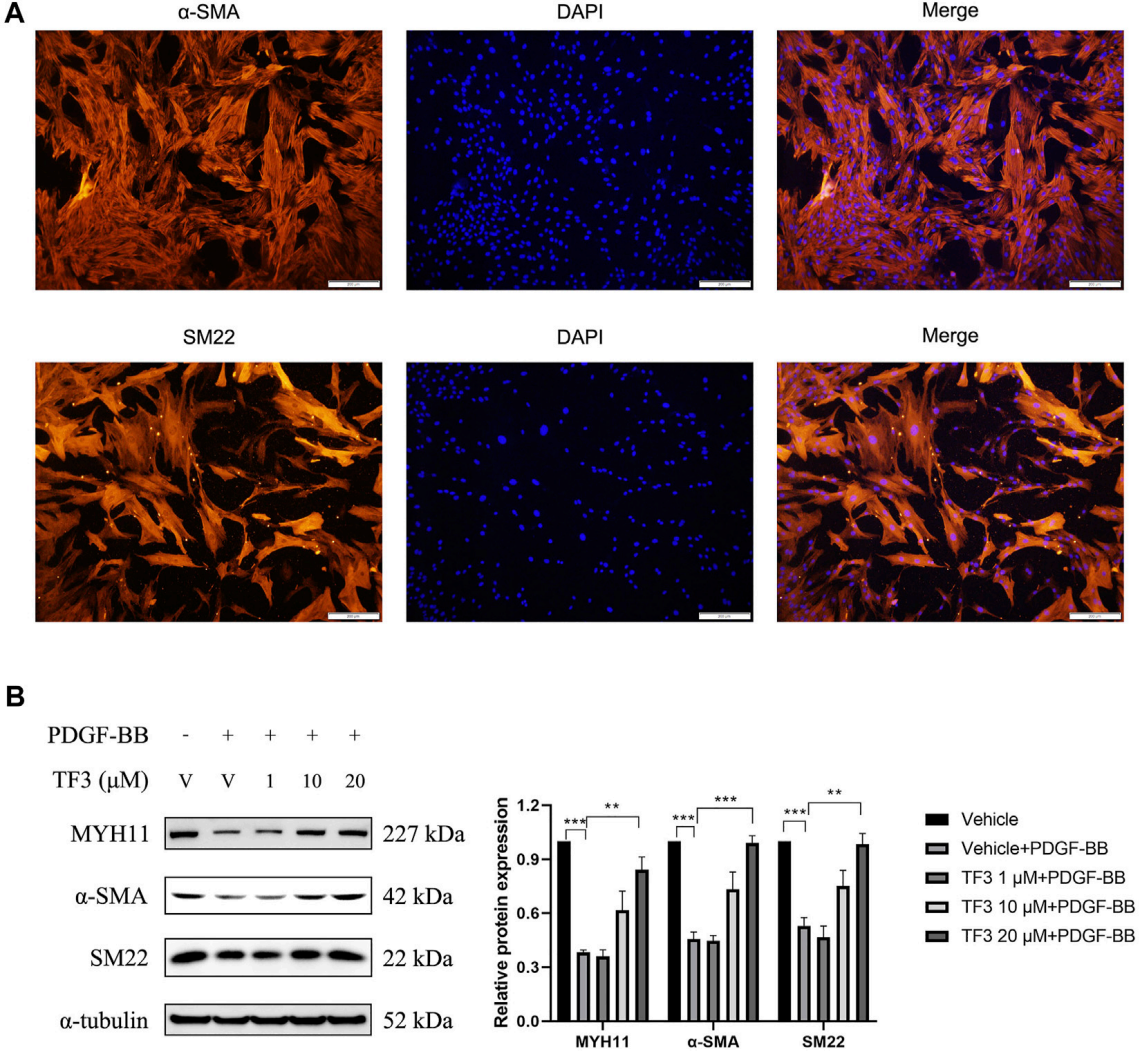
Effects of theaflavin-3,3′-digallate (TF3) on PDGF-BB–induced phenotypic switching in RASMCs. **(A)** RASMCs were stained with α-SMA (red) and DAPI (blue). Scale bar, 200 μm **(B)** The serum-starved RASMCs were pretreated with 1, 10, or 20-μM TF3 for 1 h, and then, the cells were stimulated with 20 ng/ml PDGF-BB for 48 h. The levels of contractile proteins MYH11, α-SMA, and SM22 were detected using western blot. Each experiment was performed in triplicate. **p < 0.01, ***p < 0.001.

Phenotypic switching of VSMCs is a main character of vascular restenosis after PCI. First, immunofluorescence staining of α-SMA and SM22 was used to identify the phenotype of the RASMCs, as shown in Figure 3A, and then, to evaluate the cytotoxicity of TF3 for RASMCs, MTT assay was conducted to test the cell viability. As shown in Supplementary Figure S3, TF3 was nontoxic for RASMCs at concentrations of at least 80 μM. Therefore, we selected the safety concentrations of 1, 10, and 20 μM for further experiments. Western blot was conducted to investigate whether TF3 is able to reverse PDGF-BB–induced phenotypic switching in RASMCs. The results showed that the protein expressions of MYH11, α-SMA, and SM22, which were recognized to be the contractile markers of VSMCs, were reduced after PDGF-BB (20 ng/ml) stimulation, and pretreatment of 1, 10, and 20 μM TF3 could reverse this phenomenon in a concentration-dependent manner (Figure 3B).

### TF3 Inhibits PDGF-BB–Induced RASMC Proliferation

**FIGURE 4:**
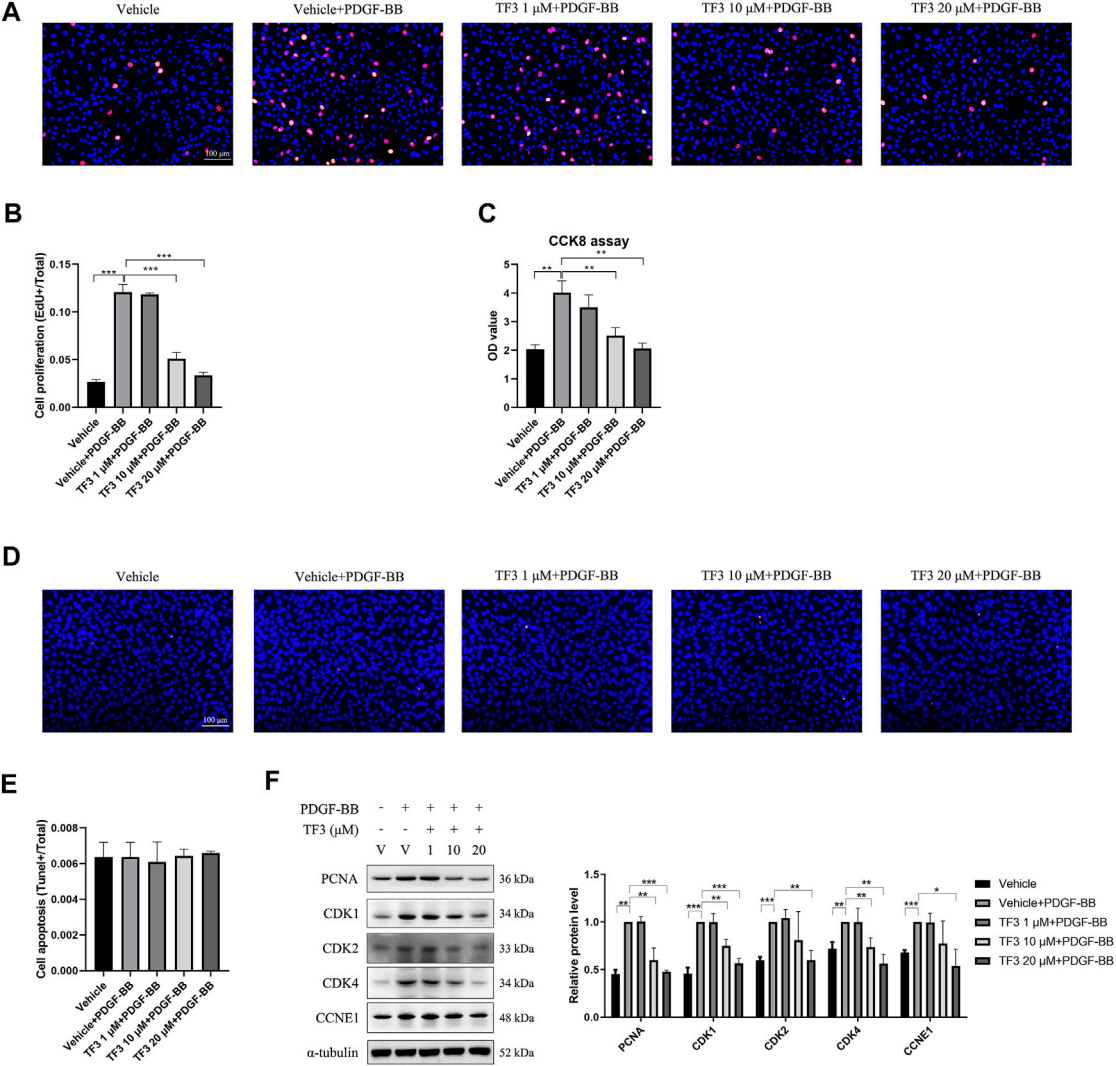
Effects of theaflavin-3,3′-digallate (TF3) on PDGF-BB–induced proliferation in rat aortic smooth cells (RASMCs). Serum-starved RASMCs were pretreated with 1-, 10-, or 20-μM TF3 for 1 h and then stimulated with 20 ng/ml PDGF-BB for 48 h. **(A)** An EdU assay was performed to evaluate the proliferation of RASMCs. **(B)** Statistical data of the EdU assay are shown. **(C)** The effect of TF3 on PDGF-BB–induced proliferation of RASMCs was also measured using CCK-8 assay. **(D)** The effect of TF3 on PDGF-BB–induced apoptosis of RASMCs was measured using terminal deoxynucleotidyl transferase dUTP nick-end labeling (TUNEL) assay. **(E)** Statistical data from the TUNEL assay are shown. **(F)** The levels of the cell proliferation-associated proteins PCNA, CDK1, CDK2, CDK4, and CCNE1 were detected by western blotting. The western blot data are shown. Each experiment was performed in triplicate. Scale bar, 100 μm, *p < 0.05, **p < 0.01, ***p < 0.001.

One of the features of neointimal hyperplasia is the abnormal proliferation and migration of VSMCs toward the intima layer. Therefore, we tested the antiproliferative effect of TF3 on PDGF-BB-stimulated RASMCs. EdU assay showed that the proliferation rate of RASMCs was increased after stimulation with PDGF-BB (20 ng/ml) for 48 h compared with the nontreated control, and pretreatment with TF3 suppressed this phenomenon in a concentration-dependent manner (Figures 4A,B). A CCK-8 assay was performed to further confirm this antiproliferative effect, in which the cells pretreated with TF3 showed much less proliferative capacity than the PDGF-BB stimulation group (Figure 4C). The terminal deoxynucleotidyl transferase dUTP nick-end labeling (TUNEL) assay showed that PDGF-BB and TF3 had no effect on cell apoptosis (Figures 4D,E). The results of western blot provided more evidence that the expressions of PCNA, CDK1, CDK2, CDK4, and CCNE1 were increased after stimulation with PDGF-BB, and pretreatment with TF3 suppressed them in a concentration-dependent manner (Figure 4F).

### TF3 Inhibits PDGF-BB–Induced RASMC Migration

**FIGURE 5:**
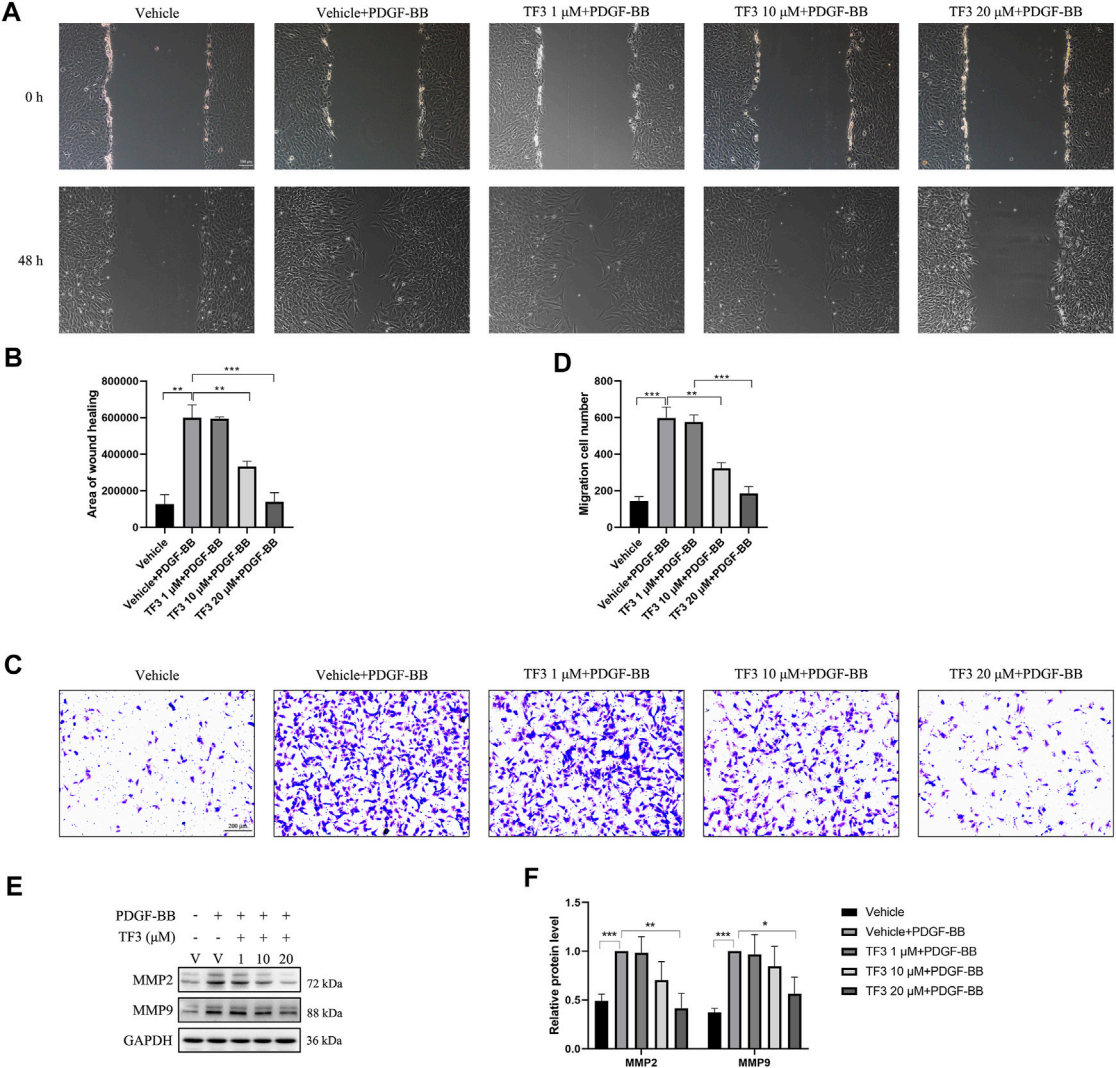
Effects of theaflavin-3,3′-digallate (TF3) on PDGF-BB–induced migration in rat aortic smooth cells (RASMCs). Serum-starved RASMCs were pretreated with 1-, 10-, or 20-μM TF3 for 1 h and then stimulated with 20 ng/ml PDGF-BB for 48 h. **(A)** The wound healing migration assay was performed as indicated. Scale bar, 100 μm **(B)** The statistical data of the wound healing assay are shown. **(C)** The transwell assay was performed as indicated. Scale bar, 200 μm **(D)** The statistical data of the transwell assay are shown. **(E)** The levels of the cell migration-associated proteins MMP2 and MMP9 were detected by western blot. **(F)** The statistical data of the western blot analysis are shown. Each experiment was performed in triplicate. *p < 0.05, **p < 0.01, ***p < 0.001.

To evaluate the effect of TF3 on PDGF-BB–induced RASMC migration, we performed a wound healing migration assay. As shown in Figures 5A,B, the cells migrated to near confluence after 48 h of PDGF-BB stimulation, and pretreatment with TF3 inhibited PDGF-BB–induced migration in a concentration-dependent manner compared with the PDGF-BB stimulation group. As expected, the transwell assay and western blot analysis showed consistent results to further prove that TF3 prevents RASMC migration under PDGF-BB induction (Figures 5C–F).

### TF3 Suppresses the Activation of PDGFRβ and Its Downstream Pathways in PDGF-BB–Induced RASMCs

PDGF-BB can bind to both PDGFRα and PDGFRβ on the surface of the cell membrane. Since the expression of β-receptors is approximately 10-fold higher than that of α-receptors (Bornfeldt et al., 1995), we tested whether TF3 targets PDGFRβ and its downstream pathways. Upon stimulation of the receptor, a series of tyrosine residues in the receptor subunits were phosphorylated. Next, ligands, such as phospholipase C-gamma 1 (PLC-γ1) and the Src family, bound with the receptor through specific phosphotyrosine residues (Claesson-Welsh, 1994) and were subsequently phosphorylated. In addition, it has been previously shown that PDGF-BB promotes RASMC proliferation and migration through activation of the PI3K/Akt/mTOR and classic MAPK pathways, the inhibition of which can postpone the above progression (Song et al., 2016; Fairaq et al., 2017; Zhang et al., 2018).

**FIGURE 6:**
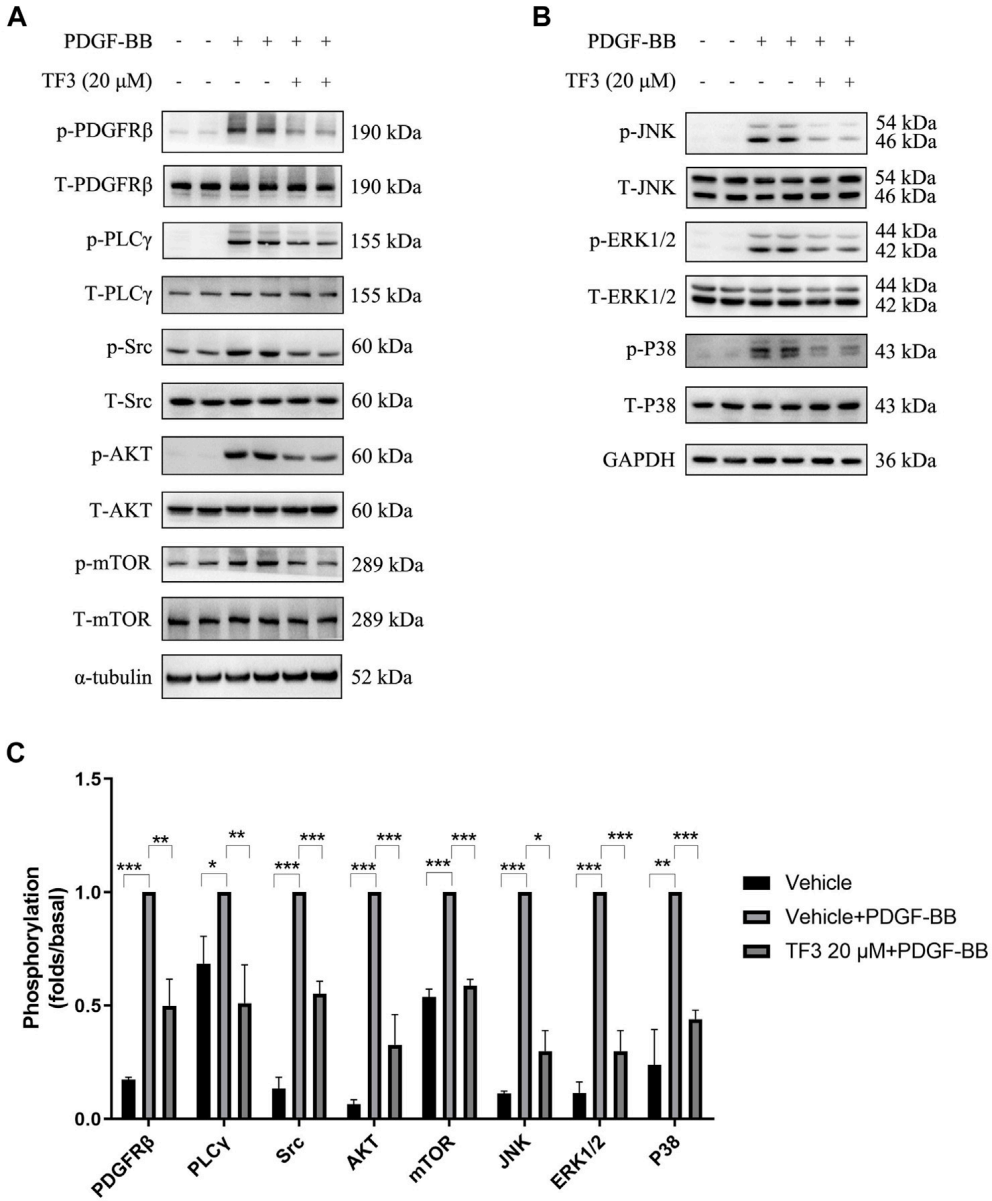
Theaflavin-3,3′-digallate (TF3) suppresses the activation of PDGFRβ and its downstream pathways in PDGF-BB–induced rat aortic smooth cells (RASMCs). The cells were serum-starved for 24 h and then stimulated with PDGF-BB (20 ng/ml) with or without pretreatment with 20 μM TF3. Cell extract was harvested after 10 min of stimulation. **(A,B)** Western blotting was performed using antibodies against the classical PDGFRβ and MAPK signaling pathways. **(C)** The statistical data of the western blot are shown. Each experiment was performed in triplicate. *p < 0.05, **p < 0.01, ***p < 0.001.

Our experiments confirmed the time-dependent manner of PDGF-BB-triggered activation in the abovementioned pathways (data not shown), and we chose 10 min as the time point to evaluate the extent of phosphorylation. Western blotting was conducted using antibodies against the classical PDGFRβ and MAPK signaling pathway components after 10 min of PDGF-BB stimulation. Under the premise that the total amount of these proteins remained the same, the expressions of p-PDGFRβ (Tyr751), p-PLCγ (Tyr783), p-Src (Tyr416), p-AKT (Ser473), p-mTOR (Ser2448), p-JNK (Thr183/Tyr185), p-ERK1/2 (Tyr204), and p-P38 (Thr180/Tyr182) were found to be increased after PDGF-BB stimulation, and pretreatment with 20 μM TF3 obviously diminished the phosphorylation of these proteins after PDGF-BB stimulation (Figure 6).

**FIGURE 7:**
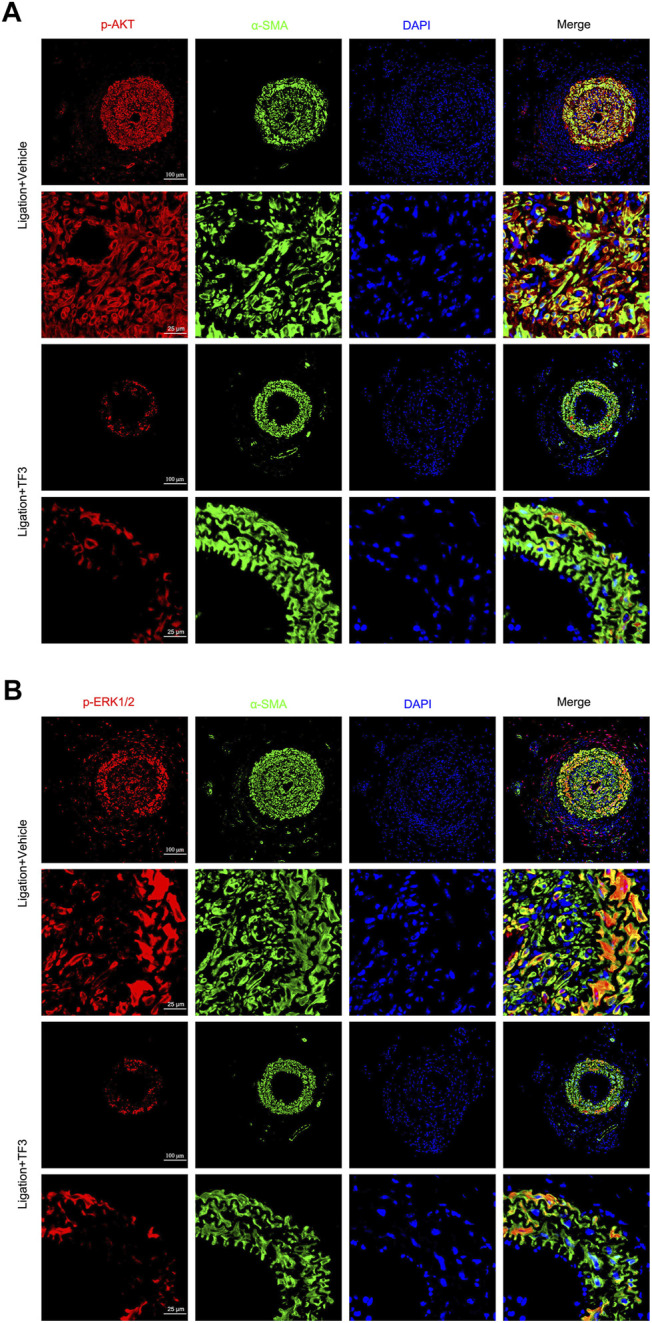
Theaflavin-3,3′-digallate (TF3) suppresses the activation of AKT and ERK1/2 during carotid artery ligation–induced neointimal hyperplasia. TF3 (10 mg/kg) or vehicle was intraperitoneally injected after carotid artery ligation. **(A)** Immunofluorescence staining of p-AKT (red), α-SMA (green), and DAPI (blue) (above) and their enlarged view (below) are shown. **(B)** Immunofluorescence staining of p-ERK (red), α-SMA (green), and DAPI (blue) (above) and their enlarged view (below) are shown. Scale bars, 100 and 25 μm.

**FIGURE 8:**
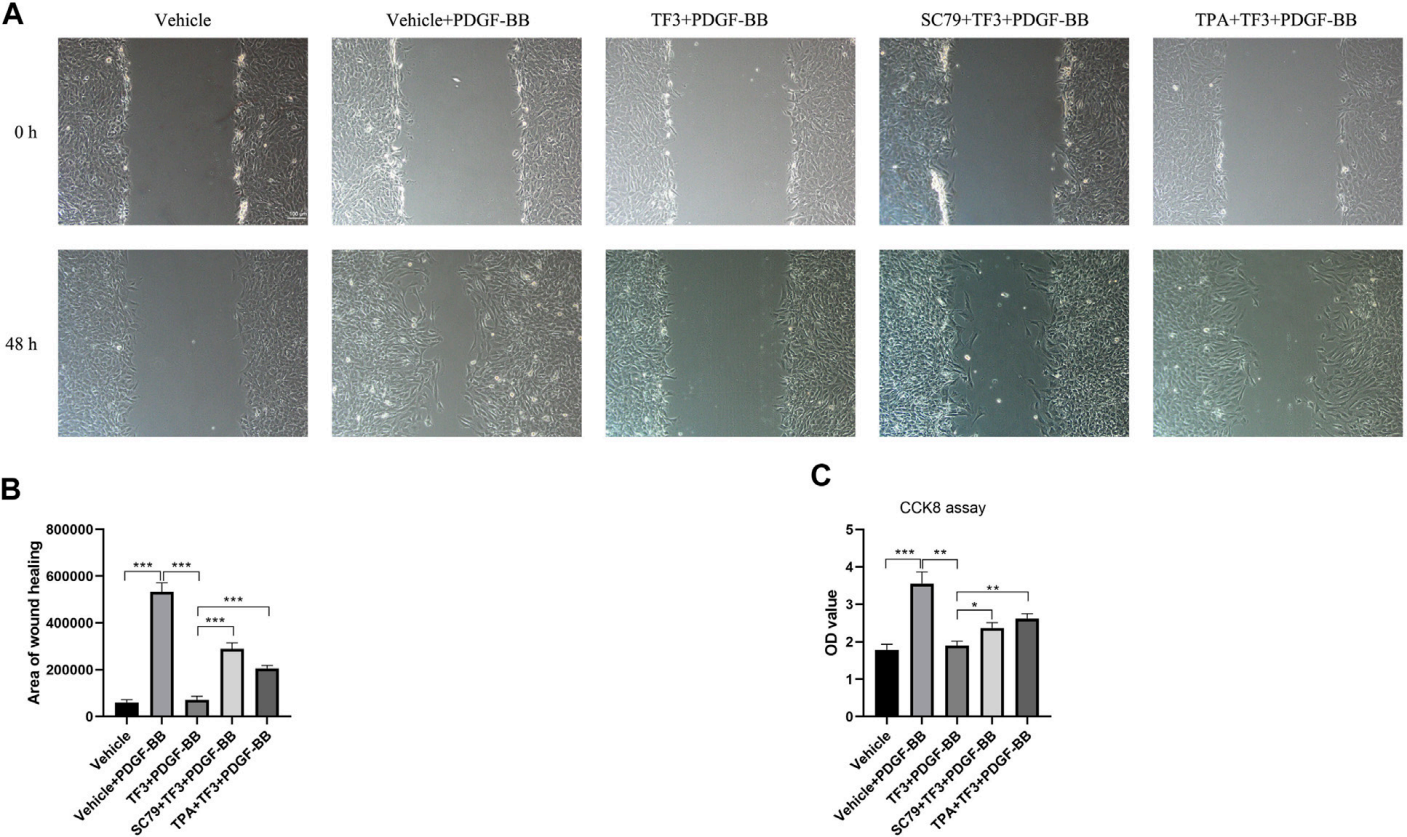
Activation of AKT and ERK1/2 abrogate the effects of theaflavin-3,3′-digallate (TF3) on rat aortic smooth cells (RASMCs) under PDGF-BB stimulation. Serum-starved RASMCs were pretreated with 20-μM TF3, 5 ug/ml SC79, and 150 nM TPA for 1 h and then stimulated with 20 ng/ml PDGF-BB for 48 h. **(A)** The wound healing migration assay was performed as indicated. Scale bar, 100 μm **(B)** The statistical data of the wound healing assay is shown. **(C)** The CCK-8 assay was performed as indicated. Each experiment was performed in triplicate. *p < 0.05, **p < 0.01, ***p < 0.001.

In order to further explore whether TF3 has the same effect *in vivo*, we conducted immunofluorescence assay at 14 days after CAL, and a strong reduction of p-AKT and p-ERK1/2 was detected under TF3 injection (Figure 7 and Supplementary Figure S4). In addition, using AKT activator SC79 (Wen et al., 2020) (Selleck, S7863) and ERK1/2 activator TPA (Xiao et al., 2019) (Shanghai yuanye Bio-Technology, B50767), we conducted CCK-8 and wound healing assay *in vitro*. Results revealed that pretreatment with SC79 (5 ug/ml) or TPA (150 nM) reversed the inhibition effects on cell proliferation and migration by TF3 under PDGF-BB stimulation to some extent (Figure 8). These results indicated that TF3 suppresses the activation of PDGFRβ and its downstream pathways during PDGF-BB stimulation to perform its pronounced effect on maintaining the contractile phenotype of RASMCs both *in vivo* and *in vitro*.

## Discussion

In the present study, we delineated that TF3 ameliorates neointimal hyperplasia *in vivo* to a large extent. Intraperitoneal injection of TF3 significantly reduced the I/M ratio after CAL as well as maintained the vessel structure. The expression levels of PCNA and MMP9 were decreased in the vascular media and intima after TF3 intervention.

Unlike cardiac or skeletal muscle cells, VSMCs are highly versatile in response to the environment change in vessel walls. Contractile VSMCs regulate the structure of blood vessels under normal conditions. In case of vascular injury, they undergo phenotypic changes from the quiescent contractile phenotype to the proliferative and migratory synthetic phenotype. PDGF-BB was found to have a key role in the modulation of this conversion (Xu et al., 2009) as it was sharply elevated in the initiation stage during neointimal hyperplasia and promoted the accumulation of VSMCs after balloon injury. Therefore, we conducted *in vitro* experiments on RASMCs using PDGF-BB as stimulation. Our results demonstrated that the expression of the contractile factors (MYH11, α-SMA, and SM22) of RASMCs were reduced after PDGF-BB stimulation, and pretreatment of TF3 can reverse this phenotypic switching to a large extent. While converting to the synthetic phenotype, RASMCs are excessively capable of proliferating and migrating as well as secreting various extracellular matrix proteins and cytokines (An et al., 2017). To observe the proliferative activity, we performed CCK8 and EdU assays. According to the results, the enhanced proliferative ability after PDGF-BB stimuli can be suppressed by TF3 to a large extent. Consistent with this finding, western blotting revealed a great decrease in the levels of some proliferation-associated proteins (PCNA, CDK1, CDK2, CDK4, and CCNE1), and TUNEL staining showed that there was apparently no increase in cell apoptosis. Moreover, wound healing and transwell assays further demonstrated the ability of TF3 to inhibit VSMC migration. Cells pretreated with TF3 exhibited attenuated migratory ability compared to the control group, and western blot analysis later confirmed the decreased level of some migration-associated proteins (MMP2 and MMP9). Therefore, we verified that TF3 is an active inhibitor of the contractile-to-synthetic phenotype switch.

Consistent with previous studies, PDGF-BB triggers the phenotypic switching of RASMCs by binding and activating PDGFRβ; and molecules, such as PLCγ1 and the Src family, are subsequently bound to the receptor and phosphorylated. In addition, the PI3K/Akt/mTOR and MAPK pathways are also involved in PDGF-BB–induced phenotypic switching (Shao et al., 2020; Osman et al., 2021). Western blot analysis confirmed the explicit inhibitory effect of TF3 on the phosphorylation of p-PDGFRβ, p-PLCγ, p-Src, p-AKT, p-mTOR, p-JNK, p-ERK1/2, and p-P38 after 10 min of stimulation. It should be noted that phosphorylation levels of p-PDGFRβ, p-AKT, and p-ERK1/2 were also downregulated by TF3 in primary mSMCs (Supplementary Figure S5). In line with *in vitro* results, activation of p-AKT and p-ERK1/2 pathways were also suppressed by TF3 *in vivo*. SC79 and TPA, activators of AKT and ERK1/2, respectively, abrogated the effects of TF3 on inhibiting RASMC phenotypic switching. Taken together, these results proved that TF3 suppresses the activation of PDGFRβ and its downstream pathways under PDGF-BB stimulation.

**FIGURE 9:**
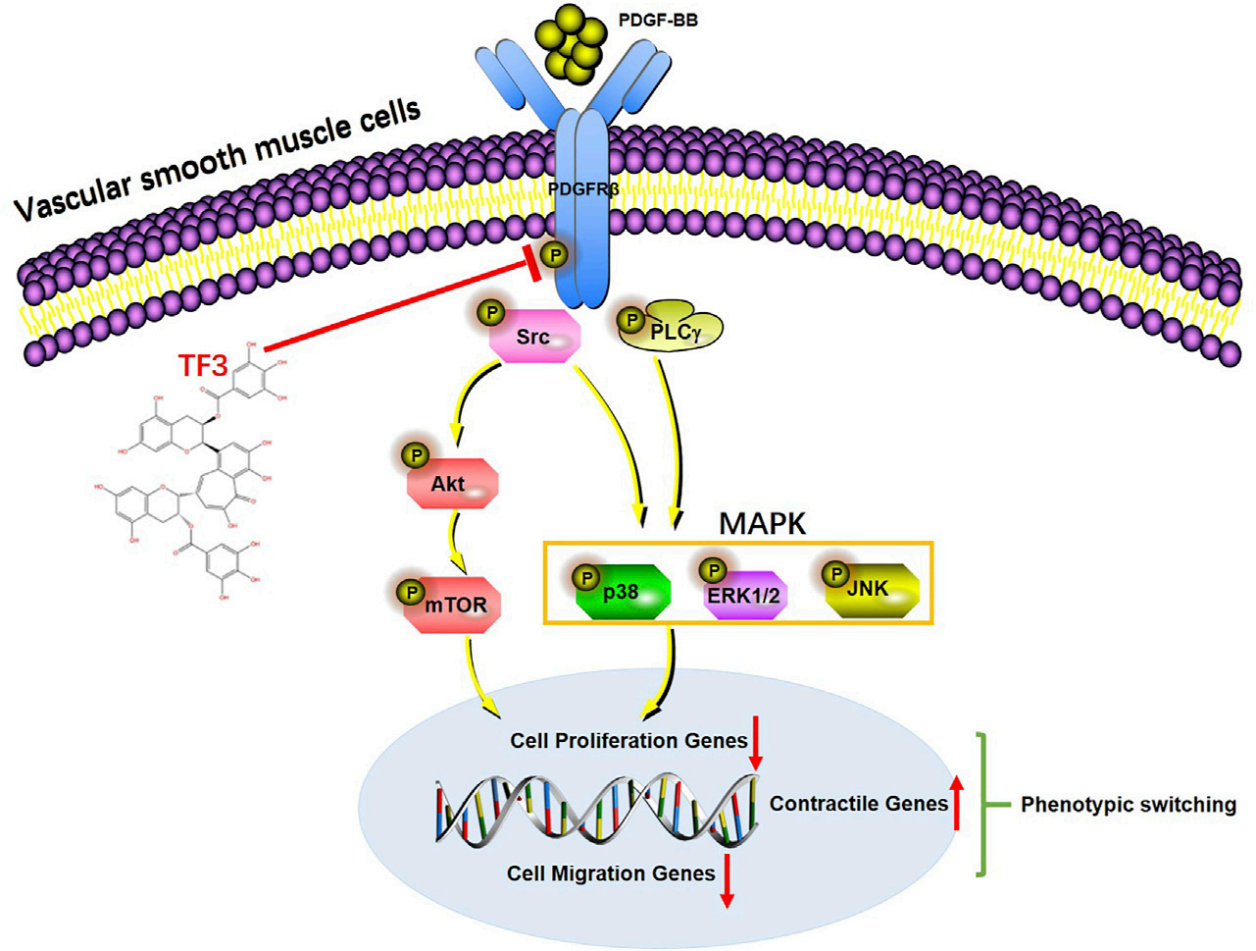
A working model of the effect of theaflavin-3,3′-digallate (TF3) in PDGF-BB–induced phenotypic switching. Under vessel injury, vascular smooth cells (VSMCs) undergo phenotypic switching that leads to neointimal hyperplasia. TF3 inhibits the activation of PDGFRβ and its downstream pathways to suppress PDGF-BB–induced VSMC phenotypic switching and thus reduces neointimal hyperplasia.

In summary, we demonstrated that TF3 acts as a key repressor of PDGF-BB–induced VSMC phenotypic switching by inhibiting the activation of PDGFRβ and its downstream pathways (Figure 9). These findings suggest that intervention with TF3 may prevent some proliferative vascular diseases, such as neointimal hyperplasia, after percutaneous coronary intervention and serve as a potential therapeutic candidate for controlling the abnormal phenotypic switching of VSMCs.

## Data Availability

The original contributions presented in the study are included in the article/Supplementary Material, and further inquiries can be directed to the corresponding authors.
