# Supplementary figures and images for: Theaflavin-3,3′-Digallate from Black Tea Inhibits Neointima Formation Through Suppression of the PDGFRβ Pathway in Vascular Smooth Muscle Cells
Source: Front Pharmacol. 2022 Jul 12;13:861319. doi: 10.3389/fphar.2022.861319 (PMC9315285; doi:10.3389/fphar.2022.861319)

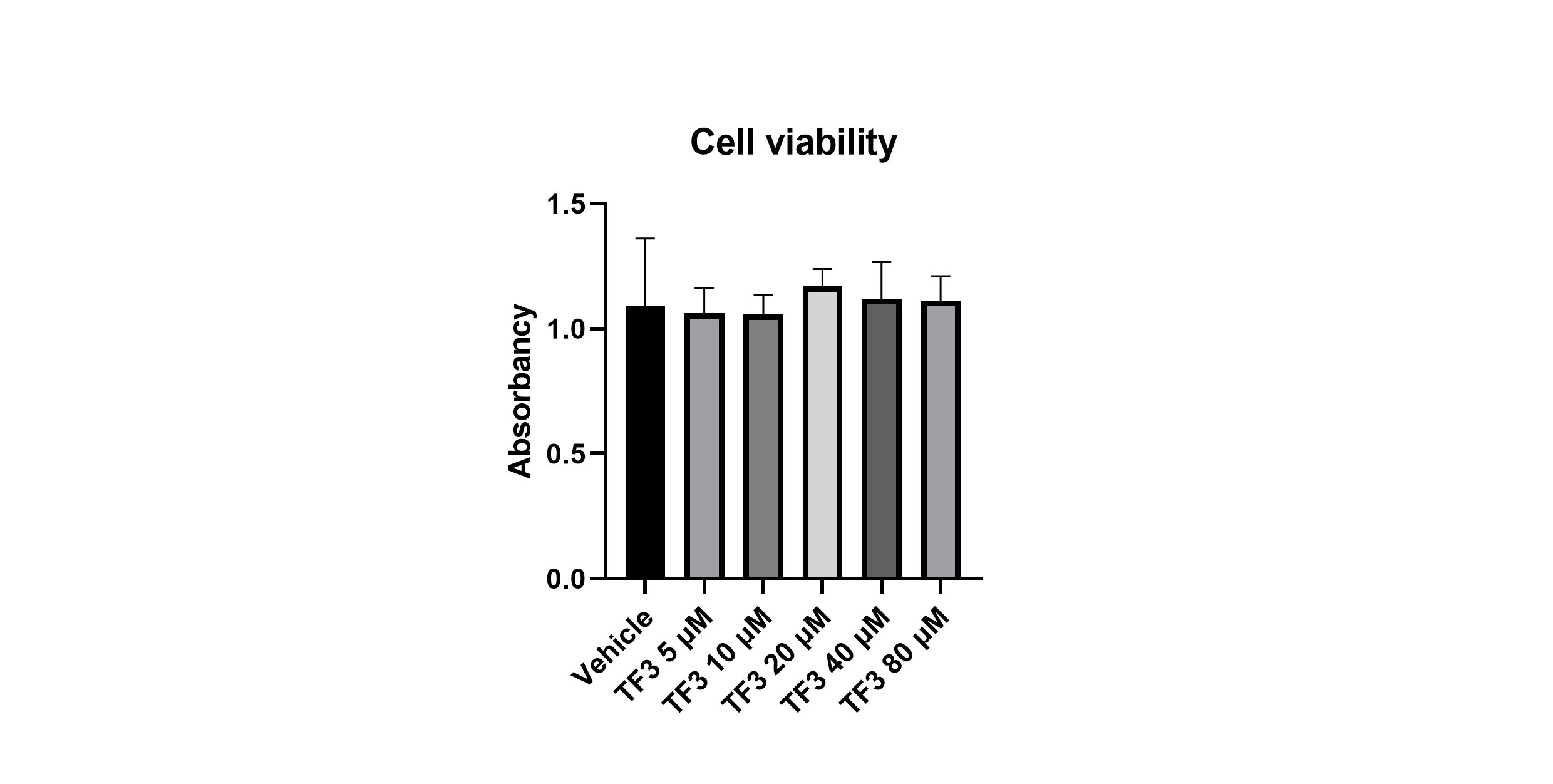

Supplement: Supplementary file 1 [file Image3.JPEG]

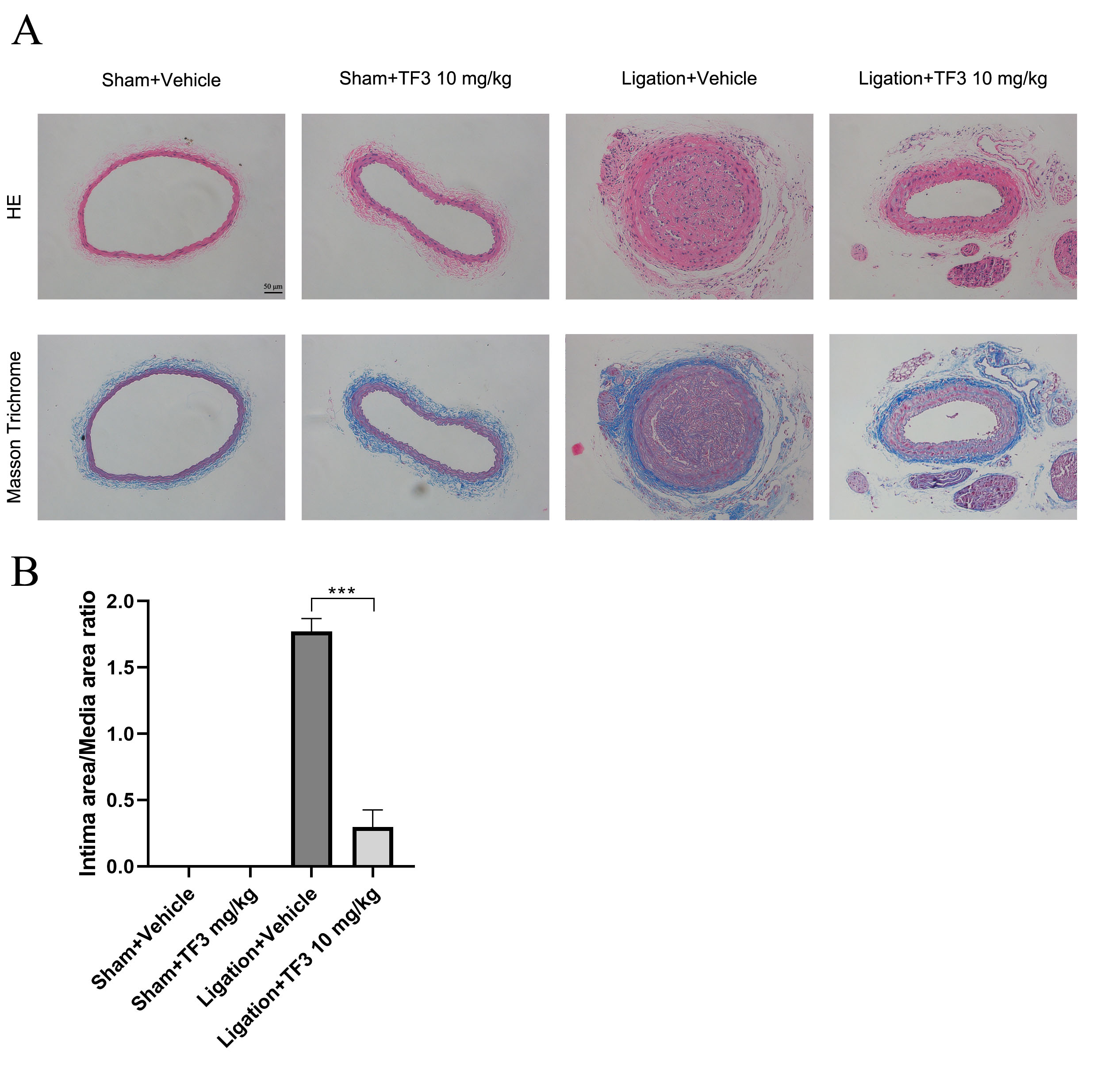

Supplement: Supplementary file 2 [file Image1.JPEG]

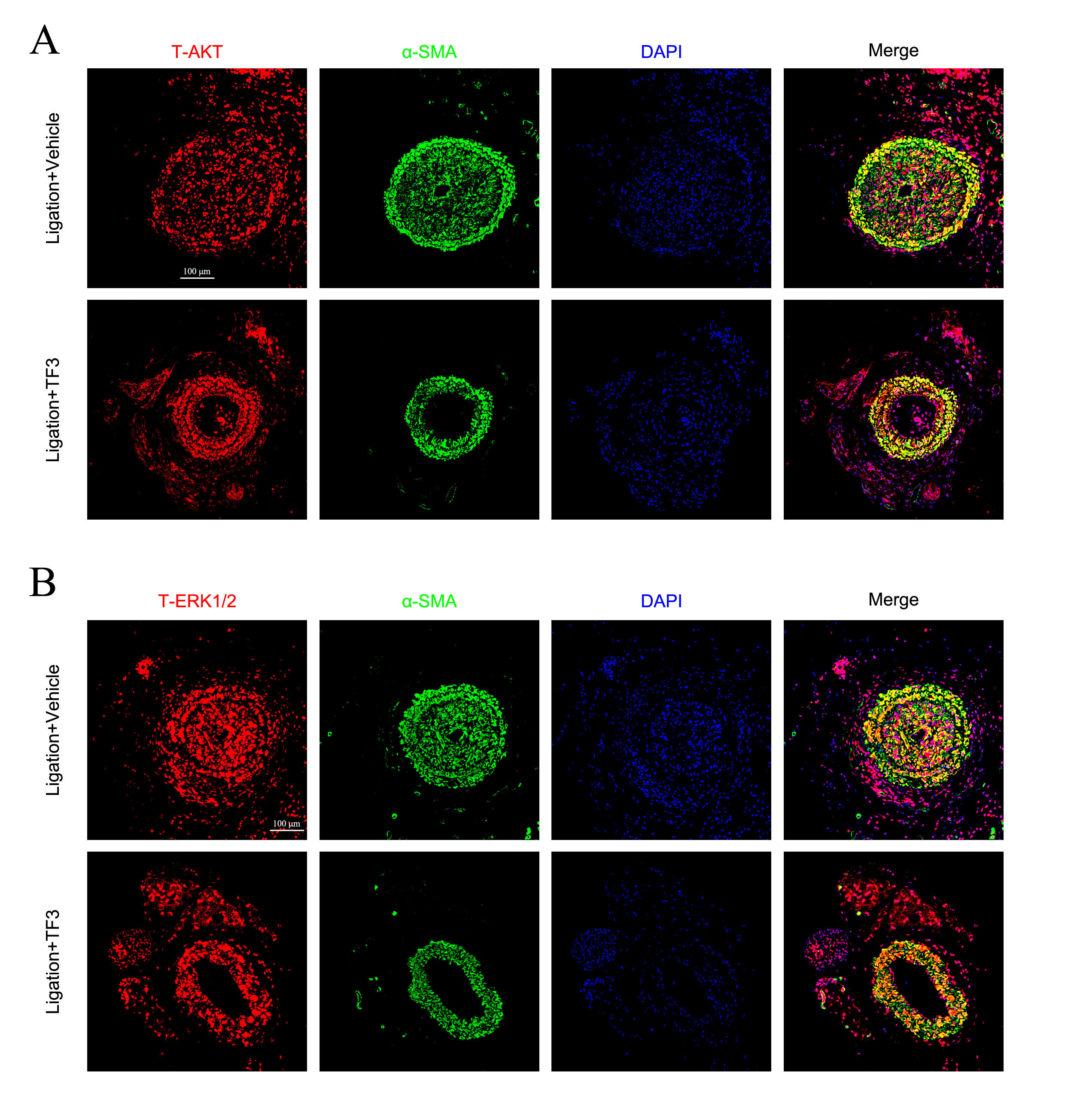

Supplement: Supplementary file 3 [file Image4.JPEG]

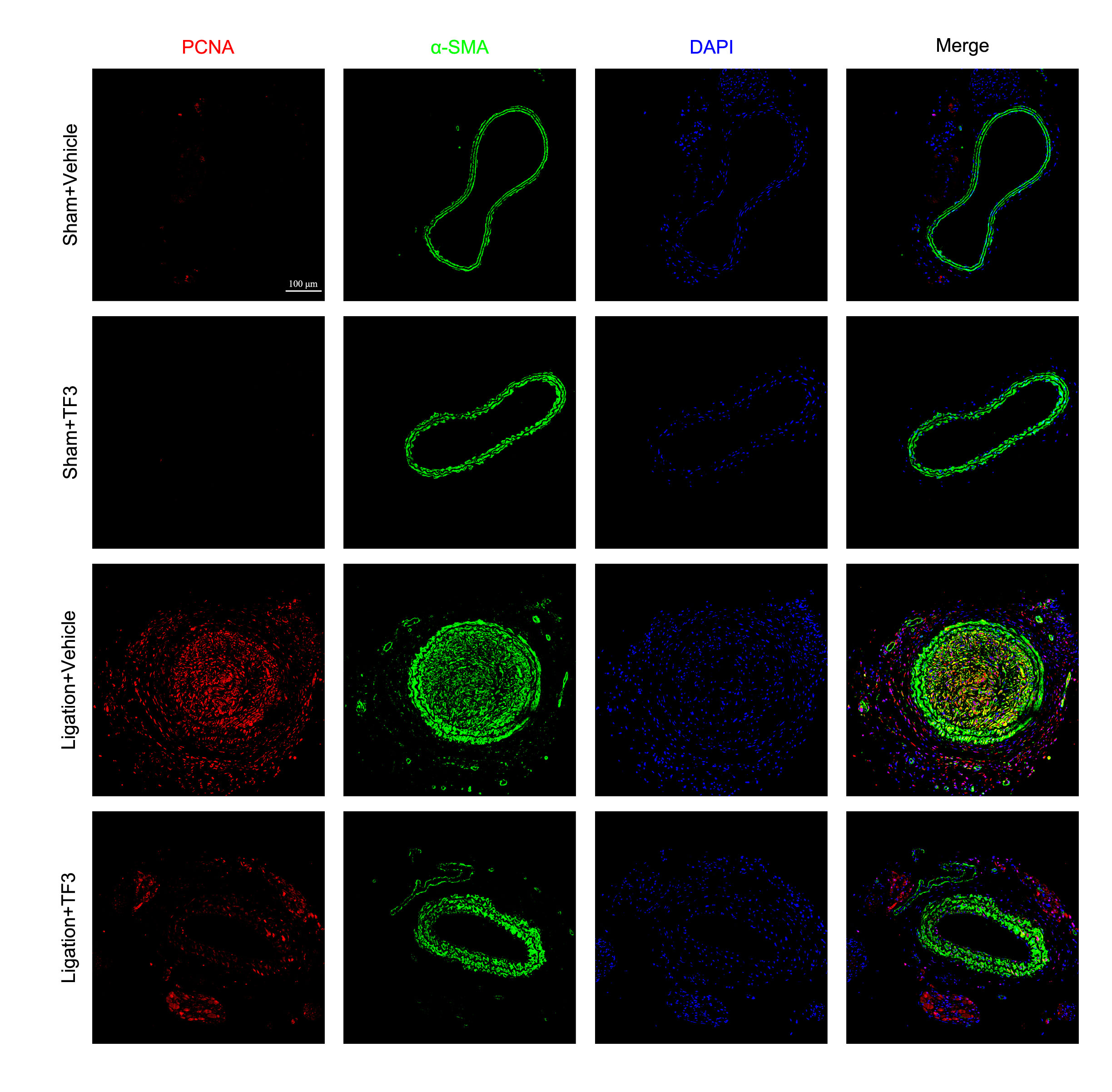

Supplement: Supplementary file 4 [file Image2.JPEG]

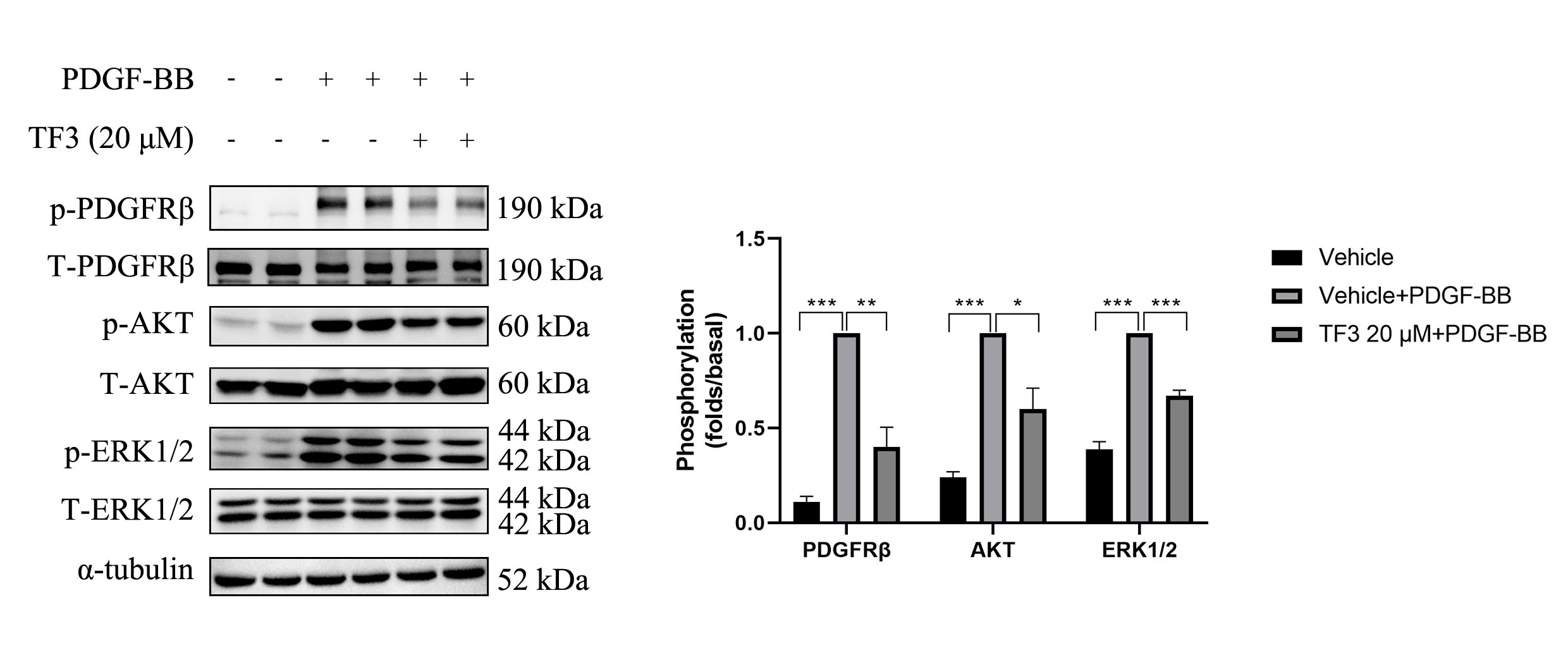

Supplement: Supplementary file 5 [file Image5.JPEG]
