# Supplementary material for: Theaflavin-3,3′-Digallate from Black Tea Inhibits Neointima Formation Through Suppression of the PDGFRβ Pathway in Vascular Smooth Muscle Cells
Source: Front Pharmacol. 2022 Jul 12;13:861319. doi: 10.3389/fphar.2022.861319 (PMC9315285; doi:10.3389/fphar.2022.861319)
Supplement: Supplementary file 6 [file DataSheet1.pdf]

Materials and Methods

MTT cell viability assay

To estimate the cell viability and proliferation, RASMCs were seeded onto 96-well plates（5000/well）and cultured in DMEM containing 10% FBS to near confluence. RASMCs were then cultured with FBS free DMEM for 48 h, and then treated with vehicle and different concentrations of TF3 (Shanghai Yuanye) from 5 μM to 80 μM for cell viability assay. Next, 50 μL MTT solution was added to each well and incubated for another 4 h at 37°C. Then, the medium was removed and 150 μL DMSO was added to each well. After shaking for 15 min，the absorbance was detected at 570 nm to determine the cell viability and rate of proliferation.

Supplementary Figure legends

Supplementary Fig. 1 Effect of TF3 on carotid artery ligation induced neointimal hyperplasia in mice 28 days after surgery.

(A) TF3 (10 mg/kg) or Vehicle was intraperitoneally injected after carotid artery ligation. Mice were sacrificed 28 days after the surgery. H&E and Masson trichrome stained sections of all groups are shown. Scale bar, 50 μm. (B) Quantification of the ratio of intima to media (I/M ratio), lumen diameter and lumen area. The data are presented as the means ± SEM (n=6). ***$p<0.001$.

Supplementary Fig. 2 Effect of TF3 on PCNA expression in carotid artery ligation induced neointimal hyperplasia in mice 28 days after surgery. TF3 (10 mg/kg) or Vehicle was intraperitoneally injected after carotid artery ligation. Immunofluorescence staining of PCNA (red), α-SMA (green) and DAPI (blue) were shown. Scale bar, 100 μm.

Supplementary Fig. 3 Effect of TF3 on the cell viability in RASMCs. The cytotoxicity of TF3 in RASMCs. Cells were treated with 5 μM, 10 μM, 20 μM, 40 μM or 80 μM TF3 for 48 h, the cell viability was measured by MTT assay. Each experiment was performed in triplicate.

Supplementary Fig. 4 TF3 suppresses the activation of AKT and ERK1/2 during carotid artery ligation induced neointimal hyperplasia.

TF3 (10 mg/kg) or vehicle was intraperitoneally injected after carotid artery ligation. (A) Immunofluorescence staining of T-AKT (red), α-SMA (green) and DAPI (blue) (above) were shown. (B) Immunofluorescence staining of T-ERK (red), α-SMA (green) and DAPI (Blue) (above) were shown. Scale bar, 100 μm.

Supplementary Fig. 5 TF3 suppresses the activation of PDGFRβ and its downstream pathways in PDGF-BB-induced mSMCs.

The cells were serum-starved for 24 h and then stimulated with PDGF-BB (GenScript, Z03572, 20 ng/ml) with or without pretreatment with different concentrations of TF3. Cell extract was harvested after 10 min of stimulation. (A) Representative Western blot analysis of phosphorylated (p-) and total (t-) PDGFRβ, AKT and ERK1/2 expression. The statistical data of the western blot are shown. Each experiment was performed in triplicate. *$p < 0.05$, ** $p<0.01$, *** $p<0.001$.
